# Reanalysis of the NCCN PD-L1 companion diagnostic assay study for lung cancer in the context of PD-L1 expression findings in triple-negative breast cancer

**DOI:** 10.1186/s13058-019-1156-6

**Published:** 2019-06-13

**Authors:** David L. Rimm, Gang Han, Janis M. Taube, Eunhee S. Yi, Julia A. Bridge, Douglas B. Flieder, Robert Homer, Anja C. Roden, Fred R. Hirsch, Ignacio I. Wistuba, Lajos Pusztai

**Affiliations:** 1 Yale University, School of Medicine, New Haven, CT USA; 2 Texas A&M, College Station, TX USA; 3 Johns Hopkins University School of Medicine, Baltimore, MD USA; 4 Mayo Clinic, Rochester, MN USA; 5 University of Nebraska Medical Center, Omaha, NE USA; 6 Fox Chase Cancer Center, Philadelphia, PA USA; 7 VA Connecticut HealthCare System, West Haven, CT USA; 8 University of Colorado Anschutz Medical Campus, Aurora, CO USA; 9 The University of Texas MD Anderson Cancer Center, Houston, TX USA; 10 Department of Pathology, Yale Pathology Tissue Services, Yale University School of Medicine, 310 Cedar St. BML 116, PO Box 208023, New Haven, CT 06520-8023 USA

**Keywords:** Non-small cell lung cancer, PD-L1, Immunohistochemistry, Triple-negative breast cancer, Atezolizumab

## Abstract

The companion diagnostic test for checkpoint inhibitor immune therapy is an immunohistochemical test for PD-L1. The test has been shown to be reproducible for expression in tumor cells, but not in immune cells. Immune cells were used in the IMpassion130 trial which showed PD-L1 expression was associated with a better outcome. Two large studies have been done assessing immune cell PD-L1 expression in lung cancer. Here, we reanalyze one of those studies, to show that, even with an easier scoring method, there is still only poor agreement between assays and pathologist for immune cell PD-L1 expression.

Companion diagnostic testing has gained increased importance of the last few years. The earliest companion tests were immunohistochemistry (IHC) based (estrogen receptor and HER2). These have recently been followed by a series of molecular, mutation-based tests (EGFR and BRAF) and most recently, another IHC test for PD-L1. When the FDA clears or approves companion diagnostic tests, it is widely assumed that these tests are accurate, reproducible, and robust. In fact, the SSED (Summary of Safety and Effectiveness Documents) released by the FDA provide the evidence to justify the assumption that the tests are worthy of consumer, payer, and physician confidence. Examination of the SSEDs for the PD-L1 tests shows that the FDA clears assays after review by only 2 or 3 pathologists, often showing high overall percent agreement (OPA) that may not reflect real-world outcomes. In fact, when PD-L1 assays were assessed by multiple observers, some FDA-approved categories were found to be unreproducible, specifically including immune cell expression of PD-L1 [1, 2].

In October of 2018, Schmid and colleagues from Genentech reported the results of the IMpassion 130 trial in first-line metastatic setting in breast cancer [3]. In a trial of atezolizumab or placebo in combination with paclitaxel, this work showed statistically significant extension of median disease-free overall survival from 15.5 to 25 months in patients with “PD-L1 positive” tumors and no benefit in PD-L1 negative tumors. While this is exciting for breast cancer patients, it is a challenge for pathologists and oncologists. Pathologists are responsible for PD-L1 status determination and the approach used in this breast cancer study conflicts with previous efforts in lung, gastric, head and neck, and cervical cancer. The standard PD-L1 expression test for atezolizumab is the Ventana SP142 assay which has been shown to have lower sensitivity than other PD-L1 assays in many studies [1, 2, 4, 5]. As such, it is impossible to validate this accurately in the CLIA lab, since there is no comparator assay, as there is for LDTs and the other FDA assays which have been shown to be equivalent. Furthermore, in breast cancer, the assay is read as a two-category immune cell (IC) score compared to the three- or four-category IC reading that was tested in two large, multi-institutional biomarker studies in lung cancer tissue [1, 2]. Both the NCCN [1] and the Blueprint 2 [2] studies concluded that pathologists cannot accurately or reproducibly read the three- or four-category IC score, with interclass correlation coefficient (ICC) between 0.19 and 0.28.

Here, we reanalyzed the data from NCCN study [1] using the original IC readings of 13 pathologists collapsed into a two-category scale using OPA (the two categories mimic the IC scoring in the IMpassion 130 study, < 1% or > 1% immune cells). For the three categories, the OPA between the four assays is 29% but using the two-category scale, the OPA rises to 54%. Similarly, inter-pathologist OPA goes from 0% (no complete agreement between 13 pathologists on 90 slides with three-category scoring) to 18% for two-category scoring (or 67% if you exclude outlier pathologist 12 in Fig. 1). Thus, collapsing of the scoring system from three to two categories improves both assay and pathologist OPA although both remain low. For comparison, ER/PR and HER2 scores have OPAs in the 90-95% range [6, 7].

**Fig. 1:**
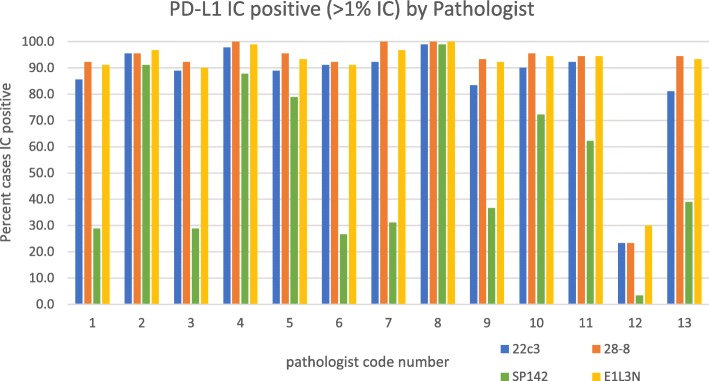
Distribution of positive binary IC score by assay

The low agreement between the assays is likely attributable to previously demonstrated lower SP142 sensitivity compared to other FDA-approved and laboratory-developed test (LDT) assays [1, 2]. It is unclear if there will be an expectation for CLIA labs already performing more sensitive PD-L1 assays, to make a switch to or an addition of the less sensitive SP142 assay for therapeutic eligibility determination. The survey data indicates that most labs are utilizing 22c3, followed by an LDT using E1L3N. To test if re-categorization of the IC component of this assay fixes this sensitivity problem, the IC scores of each NCCN study pathologist were plotted and collapsed into two categories (Fig. 1). This analysis suggests that for about one third of the pathologists, the positive/negative scoring system makes the assays equivalent, but another one third of the pathologists find dramatically fewer cases positive with the SP142 assay compared to the other assays. The variable sensitivity of the assays was unknown when the IMpassion trial began, but it would be unprecedented to have multiple assays with differential sensitivity for a single biomarker in one lab. Similarly, there is no precedent for how these variable assays could be separately standardized.

In summary, this analysis raises a significant concern for pathologists who need to provide accurate and reproducible companion diagnostic results for PD-L1. While the NCCN study data presented here are from lung cancer, not breast cancer tissue, there is no evidence that the biochemistry of the interaction has any difference between the tumor sites. While the lung cancer pathologists in the NCCN study were not trained to read IC scores, the Blueprint 2 study included 1.5 days of training for 15 pathologists and found very low concordance, suggesting that training will not solve this problem. We look forward to Genentech’s help in solving this problem. A potential solution would be a reanalysis using the SP263 assay (produced by the same vendor as the SP142 assay) or a bridging study between the SP142 assay and the SP263 assay using the IMpassion 130 tissues.

## Data Availability

Data is available from the authors on request.
